# A multicenter randomized controlled open-label trial to assess the efficacy of compound kushen injection in combination with single-agent chemotherapy in treatment of elderly patients with advanced non-small cell lung cancer: study protocol for a randomized controlled trial

**DOI:** 10.1186/s13063-016-1231-6

**Published:** 2016-03-08

**Authors:** Xue-qian Wang, Jie Liu, Hong-sheng Lin, Wei Hou

**Affiliations:** Oncology Department of Guang’anmen Hospital, Affiliated to China Academy of Chinese Medical Sciences, No. 5, Bei Xian-ge Road, Xi Cheng District Beijing, 100053 China; Beijing University of Chinese Medicine, No. 11, East Road North 3rd Ring Road, Chao Yang District Beijing, 100029 China

**Keywords:** Compound kushen injection, Elderly, Non-small cell lung cancer, Single-agent chemotherapy, Study protocol

## Abstract

**Background:**

With the aging of the global population, an increasing number of elderly are diagnosed with advanced non-small cell lung cancer. Although systematic chemotherapy has been one of the primary treatments for advanced non-small cell lung cancer worldwide, the elderly cannot always tolerate standard platinum-based doublet chemotherapy, thus resulting in treatment failure. To reduce toxicity, single-agent chemotherapy is often used to treat the elderly with non-small cell lung cancer; however, this may increase the risk of treatment failure due to an inadequate dose. It has been shown that compound kushen injection in combination with chemotherapy can enhance the efficacy and reduce the toxicity. The aim of this trial is to assess the clinical effectiveness and safety of compound kushen injection in combination with single-agent chemotherapy versus platinum-based doublet chemotherapy in the treatment of elderly patients with advanced non-small cell lung cancer.

**Methods:**

This multicenter study will be an open-label, randomized controlled trial. Three hundred seventy elderly patients with advanced non-small cell lung cancer will be randomly divided into experimental (*n* = 185) and control groups (*n* = 185) to receive compound kushen injection in combination with single-agent chemotherapy or standard platinum-based doublet chemotherapy for two cycles. After two cycles, the disease control rate, objective response rate, clinical symptoms, quality of life, Karnofsky Performance Status, and side effects will be assessed. Follow-up evaluations will be performed every 8 weeks to evaluate the progression-free and overall survival.

**Discussion:**

Before the trial was designed, compound kushen injection was shown to be effective for lung cancer through basic experiments and clinical trials. This study will determine whether or not the efficacy of compound kushen injection in combination with single-agent chemotherapy is comparable to that of platinum-based doublet chemotherapy, and whether or not the toxicity of compound kushen injection in combination with single-agent chemotherapy is lower than that of platinum-based doublet chemotherapy.

**Trial registration:**

ChiCTR-IPR-14005484 (16 November 2014).

## Background

The incidence of lung cancer has been increasing rapidly worldwide. Currently, lung cancer morbidity and mortality are greater than the morbidity and mortality of other malignant tumors [1], and lung cancer morbidity and mortality are closely related to age. According to statistical data from the United States National Cancer Institute, the morbidity and mortality of patients with lung cancer who are > 65 years of age are 9.8- and 16.5-fold greater than those of patients who are < 65 years of age, respectively [2]. Because aging of the global population is on the rise, the morbidity and mortality of lung cancer are also increasing.

Five and 10 years after the diagnosis of lung cancer, the percent survival for patients < 50 years of age is 16 % and 10 %, respectively. In contrast, the percent survival for patients > 70 years of age is 12 % and 5 %, respectively, which means that older people have a lower probability of surviving lung cancer. Using non-small cell lung cancer (NSCLC) as an example, which accounts for > 80 % of all lung cancer pathologies [3], 77.69 % of patients are > 65 years of age when a definite diagnosis is established [4]. Over the past 20 years, the mortality of NSCLC among youth has decreased, but mortality among the aged is on the rise [5].

In the treatment of patients with advanced NSCLC, systematic chemotherapy with platinum-based doublets is considered as standard therapy. Sörenson et al. [6] have demonstrated that chemotherapy consisting of platinum-based combinations for patients with advanced NSCLC can provide a survival advantage over supportive care alone. However, in addition to aging, elderly patients begin to deteriorate due to a decline in metabolism and a decay in organ function, which will result in drug clearance problems and increased chemotherapy toxicity. Thus, some older patients with advanced NSCLC cannot tolerate the side effects caused by standard chemotherapy [7]. Nevertheless, although single-agent chemotherapy induces less toxicity compared with doublet chemotherapy, single-agent chemotherapy will also reduce efficacy and may result in treatment failure. Delbaldo et al. [8] conducted a meta-analysis that pooled 65 trials (*n* = 13,601 patients). Specifically, comparing a doublet regimen with a single-agent regimen, a significant decrease was noted in tumor response (OR = 0.42, 95 % CI = 0.37–0.47, *P* < 0.001) and 1-year survival (OR = 0.80, 95 % CI = 0.70–0.91, *P* < 0.001) in favor of the single-agent regimen. Therefore, an effective therapy with low toxicity is urgently needed for the elderly with advanced lung cancer.

Integrated traditional Chinese and Western medicine therapy have been the most distinctive methods in the treatment of malignant tumors in China. In the treatment of NSCLC, traditional Chinese medicine (TCM) in combination with chemotherapy can enhance the efficacy and reduce chemotherapy-induced toxicity [9, 10]. As a representative of TCM injection, compound kushen injection (CKI) has good effects on pain relief, ensuring hemostasis, anti-inflammatory activity, and anti-fibrosis [11]. CKI also has efficacy in the treatment of lung cancer [12]. When combined with chemotherapy, CKI enhances efficacy, reduces toxicity, and protects organs to improve the quality of life (QoL) and help patients complete chemotherapy [13, 14]. When considering elderly patients’ characteristics, the side effects of traditional chemotherapy, and the unique function of CKI, treatment with CKI in combination with single-agent chemotherapy could be more reasonable and effective for the elderly with advanced lung cancer compared to standard chemotherapy.

Currently, large-scale clinical trials always exclude elderly patients with lung cancer [15]. As a result, there is little high-level or large-scale evidence from evidence-based medicine with a focus on the elderly with lung cancer [16]. Therefore, there is an urgent need to find a new effective therapy with less toxicity for the elderly with lung cancer. To evaluate the efficacy and safety of CKI in combination with single-agent chemotherapy for the elderly with advanced NSCLC, a multicenter, randomized, controlled, open-label trial has been designed with disease control rate (DCR) as the main observation index. A novel therapy for the elderly with advanced lung cancer is expected.

## Methods/design

### Study design

**Fig. 1:**
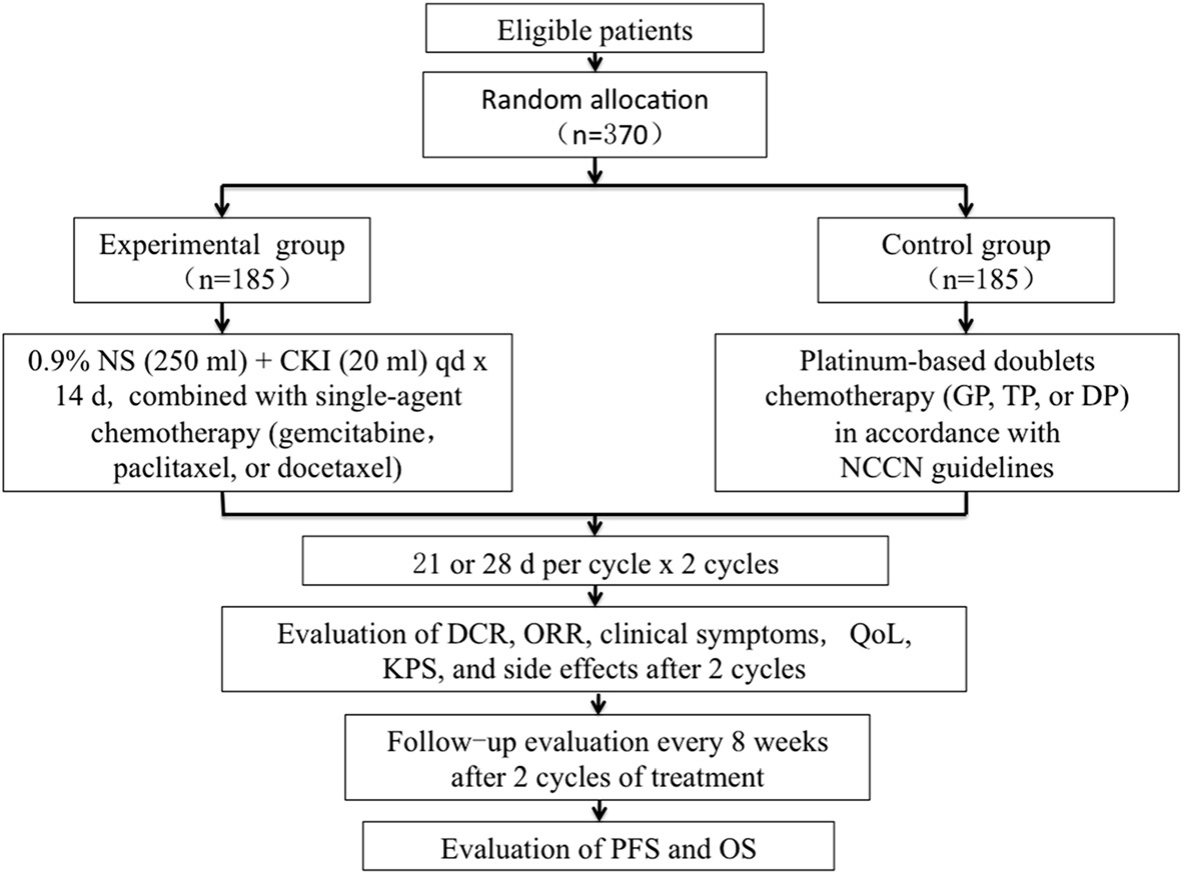
Flow diagram of the trial

This is a multicenter, randomized, controlled, open-label phase IV trial. Guang’anmen Hospital of the China Academy of Chinese Medical Sciences, TCM Hospital of Shanxi Province, TCM Hospital of Zhejiang Province, Anhui Provincial Hospital, and Tongde Hospital of Zhejiang Province will participate in this study. The included subjects (*n* = 370) will be randomly divided into experimental (*n* = 185) and control groups (*n* = 185) according to a random number table, which is generated by SAS 9.3. Both groups will undergo two treatment cycles with 21 days per cycle. The treatment course may extend to 28 days a cycle if necessary. The primary and secondary aims will be assessed after two cycles. After two cycles, follow-up evaluations will be performed every 8 weeks until death occurs. In addition, the block size and treatment assignment table will not be available to the investigators until the end of the study (Fig. 1).

### Eligibility criteria

Eligible patients will be those who meet all of the following inclusion criteria and who have none of the listed exclusion criteria.

#### Diagnostic criteria

The diagnostic criteria and TNM classification of NSCLC in this study refer to “Chinese common malignant tumor diagnosis and treatment norm—lung cancer diagnostic criteria,” as promulgated by the Ministry of Health of the People’s Republic of China.

#### Inclusion criteria

The inclusion criteria were as follows:

1. Before the start of the study, patient fully understands the study and is willing to sign the informed consent form;
2. 65–75 years of age without gender restriction;
3. Histologic or cytologic confirmation of III-IV stage NSCLC;
4. According to RECIST (version 1.1), there should be at least one measurable lesion without local treatment, and the maximum diameter should be > 10 mm (the shortest diameter of malignant lymph nodes should be > 15 mm) by spiral CT or MRI;
5. Eastern Cooperative Oncology Group (ECOG) performance status less than at least 2 points;
6. The last radiotherapy or chemotherapy session should be > 4 weeks;
7. White blood cell (WBC) count > 3.0 × 10^9^/L, neutrophil count > 1.5 × 10^9^/L, platelet (PLT) count > 60 × 10^9^/L, hemoglobin (Hb) > 8.0 g/dl, total bilirubin (TBIL) within the normal range or < 1.5 × the upper limit of normal (ULN), either aspartate transaminase (AST [SGOT]) or alanine transaminase (ALT [SGPT]) < 2.0 × ULN); and serum creatinine (SCr) < 1.5 × ULN;
8. Expected survival > 3 months.

#### Exclusion criteria

The exclusion criteria were as follows:

1. CKI within 2 weeks;
2. Only immeasurable lesions, such as pleural effusions, pulmonary lymphangitic carcinomatosis, ascites, peritoneal cancer lesions, diffuse hepatic involvement, and bone metastases;
3. Severe, uncontrolled organic lesions or infections, such as decompensated heart, lung, and kidney functional failure, leading to intolerance of chemotherapy;
4. Current participation in another clinical study or participation in another clinical study within 30 days;
5. Pregnant or lactating women;
6. Allergy to the test drug.

### Recruitment

Recruitment began in November 2014 and is expected to end in May 2016. Within 18 months after beginning the trial, patients will be screened according to the inclusion and exclusion criteria to identify eligible participants. This trial will be designed in accordance with the principle of protecting patient rights and interests, and carried out in accordance with the Declaration of Helsinki (version 8), Good Manufacturing Practice (GMP) guidelines, and the 2007 *Biological Ethics Review Method Involving Humans* by the Ministry of Health of the People’s Republic of China. Written informed consent will be obtained from all patients before participation in the trial.

### Study aims

The primary aim of this study will be to observe whether or not CKI in combination with single-agent chemotherapy has a similar or better effect on improving the DCR and objective response rate (ORR) in elderly patients with advanced NSCLC versus platinum-based doublet chemotherapy. The secondary aims of our trial will be to compare progression-free survival (PFS), overall survival (OS), clinical symptoms, quality of life (QoL), Karnofsky Performance Status (KPS), and adverse drug reactions (ADRs) between the two groups.

### Sample size

This trial will be a non-inferiority trial, in which the primary endpoint will be DCR. Schiller et al. [17] compared four chemotherapy regimens (cisplatin and paclitaxel, cisplatin and gemcitabine, cisplatin and docetaxel, and carboplatin and paclitaxel) for advanced NSCLC, in which the DCRs were 51 %, 60 %, 58 %, and 51 %, respectively. It is assumed that 60 % of DCRs in each treatment arm will respond to treatment within two cycles. A non-inferiority margin of 15 % and a one-sided type I error of 2.5 % ensured 80 % power to demonstrate non-inferiority of CKI in combination with single-agent chemotherapy versus platinum-based doublet chemotherapy. Allowing for approximately 10 % of patients to be excluded from the population, an enrollment of 370 patients (that is, 185 patients per treatment arm) is planned.

### Recruitment

The standard of care for the first-line treatment of advanced NSCLC remains the use of platinum-based doublets (GP, TP, or DP) in patients with good performance status and no significant comorbidities [18, 19]. In this trial, the DCR will serve as the primary outcome. The GP, TP, and DP regimens, which are the third-generation cytotoxic agents, have similar DCR effects on advanced NSCLC [17, 19]. Consequently, the three chemotherapy regimens were permitted to be administered at the same time. Considering the physiologic characteristics of the elderly, if the efficacy of CKI in combination with single-agent chemotherapy on elderly patients with advanced NSCLC is not inferior to platinum-based doublet chemotherapy, the long-term effects or side effects of chemotherapy are not excessive, and compound therapy is superior to platinum-based doublet chemotherapy, it seems reasonable to treat the elderly with advanced NSCLC using this therapeutic method.

### Randomization

Randomized sequences will be generated by the contract research organization (CRO), Da Wei Asian Pharmaceutical Technology (Beijing) Co., Ltd., as the third party. SAS 9.3 software will be used to generate a random sequence table for the subjects. Three hundred seventy random numbers and the allocation sequence table of the randomized group will be kept as blind codes. The subjects will be randomly divided into the experimental (*n* = 185) and control (*n* = 185) groups at a ratio of 1:1.

### Allocation concealment

According to the randomized sequences, the third party will place paper strips with black characters on a gray background into non-transparent envelopes, which will be sealed with adhesive tape.

### Intervention

Patients in the experimental group will receive mainline treatments of 250 ml of CKI (20 ml diluted in 0.9 % normal saline [0.9 % NS]), and 14 days of continuous and once daily use, which will be accompanied by single-agent chemotherapy (gemcitabine, paclitaxel, or docetaxel). Patients in the control group will receive mainline treatments of platinum-based doublet chemotherapy (GP, TP, or DP). The three regimens have no statistically significant difference in the response rates, PFS, and OS [16]. A chemotherapy cycle is 21 or 28 days in length. The outcomes will be assessed after two cycles; then the follow-up evaluations begin. CKI (specifications: 5 ml/tube) is produced by Shanxi Zhendong Pharmaceutical Co., Ltd. (Shanxi China). All chemotherapy usage will be in accordance with the National Comprehensive Cancer Network (NCCN) guidelines (Table 1).

**Table 1:** Chemotherapy regimens for NSCLC

Chemotherapy regimen	Dosage (mg/m^2^)	Medication time
TP:		
Paclitaxel	135–175	D1
Cis-platinum	75	D1
Carboplatin	AUC = 5–6	D1
GP:		
Gemcitabine	1,250	D1, D8
Cis-platinum	75	D1
Carboplatin	AUC = 5–6	D1
DP:		
Docetaxel	75	D1
Cis-platinum	75	D1
Carboplatin	AUC = 5–6	D1

*Notes*: The dosage of chemotherapy regimens are only for reference, and specific implementation will take clinical application as a standard

### Withdrawal conditions

#### Termination by researchers

Study participants may be withdrawn from the study by the researchers for the following reasons: 1) some complications and special physiologic changes may occur in subjects who are not suitable for treatment without a break; 2) disease progression is confirmed by iconography and clinical evidence; and 3) serious ADRs may occur.

### Termination by subjects

According to the informed consent form, the subjects have the right to withdraw from the trial. Subjects who do not accept treatment or re-examinations will be withdrawn. The reasons for withdrawal will be specified and recorded.

### Standards for discontinued testing

The following standards for discontinued testing will be applied: 1) a serious safety problem occurs; 2) major mistakes are found in the designed clinical protocol during the trial; and 3) the sponsor of this trial requests discontinued testing because of a lack of funds or poor management.

The withdrawal conditions should be recorded in detail for further analysis after completion of the study.

### Outcome measures and definitions

#### Primary outcome measures

DCR and ORR will be our primary outcome measures. Complete response (CR), partial response (PR), stable disease (SD), and progressive disease (PD) were defined according to the Response Evaluation Criteria in Solid Tumors (RECIST), as developed by the World Health Organization [20]. CR is defined as the disappearance of all target lesions and no new lesions. PR is defined as at least a 30 % decrease in the sum of the longest diameter of the measurable lesions. PD is defined as at least a 20 % increase in the sum of the longest diameter of the measurable lesions. SD is defined as small changes between PR and PD. DCR is the summed percentage of CR, PR, and SD in all cases, and ORR is the summed percentage of CR and PR.

### Secondary outcome measures

PFS refers to the interval of time from the beginning of the study to disease progression for the first time or patient death.

OS indicates the interval of time from the first date of medication to death for any reason.

Clinical symptoms refer to application of the M.D. Anderson Symptom Assessment Inventory [21] with 10 additional TCM items (MDASI-TCM), and it has been shown that this method of questionnaire is a reliable, valid, sensitive instrument for evaluating the TCM symptoms of cancer [22]. The newly added items of TCM symptoms include cough, expectoration, palpitation, sweating, bitter taste, oral ulcers, diarrhea, constipation, dysphoria, and feverish palms and soles. Each symptom is graded from 0–10 according to patient symptom severity, where 0 indicates no symptoms and 10 indicates the most severe level of symptoms that a patient can imagine.

QoL refers to application of the QoL scale (EORTC QLQ-C30, Chinese version) to evaluate the impact of drugs on patients with advanced NSCLC [23]. EORTC QLQ-C30 is the core scale, and includes 30 items. The method of usage is as follows: the 29th and 30th items are divided into 7 grades. According to patient answers, the items are recorded from 1–7 points. Other items are divided into 4 grades, which are recorded from 1–4 points. For application and statistical analysis, the 30 items of EORTC QLQ-C30 (Table 2) are divided into 15 domains, including 5 functional domains, 3 symptomatic domains, 1 life-quality domain, and 6 single items (each one acts as a domain). The Raw Score (RS) in every domain is the average score of all items in the domain, according to the following formula: RS = (Q1 + Q2 + „„ + Qn)/n (Table 3). To compare the scores in every field with each other, we will use the range method of linear transformation to transform RS into the standard score (SS), which is obtained from 0–100. Specifically, the calculation formula areas are as follows (R stands for the score range): ① functional domain, SS = [1-(RS-1)/R] × 100; ② symptomatic and life-quality domains, SS = [(RS-1)/R] × 100.

**Table 2:** EORTC QLQ-C30

Questions	Not at all	A little	Quite a bit	Very much
1. Do you have any trouble doing strenuous activities, like carrying a heavy shopping bag or a suitcase?	1	2	3	4
2. Do you have any trouble taking a long walk?	1	2	3	4
3. Do you have any trouble taking a short walk outside of the house?	1	2	3	4
4. Do you need to stay in bed or a chair during the day?	1	2	3	4
5. Do you need help with eating, dressing, washing yourself, or using the toilet?	1	2	3	4
During the past week:				
6. Were you limited in doing either your work or other daily activities?	1	2	3	4
7. Were you limited in pursuing your hobbies or other leisure time activities?	1	2	3	4
8. Were you short of breath?	1	2	3	4
9. Have you had pain?	1	2	3	4
10. Did you need to rest?	1	2	3	4
11. Have you had trouble sleeping?	1	2	3	4
12. Have you felt weak?	1	2	3	4
13. Have you lacked appetite?	1	2	3	4
14. Have you felt nauseated?	1	2	3	4
15. Have you vomited?	1	2	3	4
16. Have you been constipated?	1	2	3	4
During the past week:				
17. Have you had diarrhea?	1	2	3	4
18. Were you tired?	1	2	3	4
19. Did pain interfere with your daily activities?	1	2	3	4
20. Have you had difficulty in concentrating on things, like reading a newspaper or watching television?	1	2	3	4
21. Did you feel tense?	1	2	3	4
22. Did you worry?	1	2	3	4
23. Did you feel irritable?	1	2	3	4
24. Did you feel depressed?	1	2	3	4
25. Have you had difficulty remembering things?	1	2	3	4
26. Has your physical condition or medical treatment interfered with your family life?	1	2	3	4
27. Has your physical condition or medical treatment interfered with your social activities?	1	2	3	4
28. Has your physical condition or medical treatment caused you financial difficulties?	1	2	3	4
For the following questions please circle the number between 1 and 7 that best applies to you.				
29. How would you rate your overall health during the past week?				
1 2 3 4 5 6 7				
Very poor Excellent				
30. How would you rate your overall quality of life during the past week?				
1 2 3 4 5 6 7				
Very poor Excellent

**Table 3:** Calculation method for each domain in QLQ-C30 (V3.0)

Field	Code	Character	Item amount	Scores (R)	Calculation method
Physical function	PF	Function	5	3	(Q1 + Q2 + Q3 + Q4 + Q5)/5
Role function	RF	Function	2	3	(Q6 + Q7)/2
Emotional function	EF	Function	4	3	(Q21 + Q22 + Q23 + Q24)/4
Cognitive function	CF	Function	2	3	(Q20+ Q25)/2
Social function	SF	Function	2	3	(Q26 + Q27)/2
Global QOL	QL		2	6	(Q29 + Q30)/2
Fatigue	FA	Symptom	3	3	(Q10 + Q12 + Q26)/3
Nausea and vomiting	NV	Symptom	2	3	(Q14 + Q15)/2
Pain	PA	Symptom	2	3	(Q9 + Q19)/2
Dyspnea	DY	Symptom	1	3	Q8
Sleep disturbance	SL	Symptom	1	3	Q11
Appetite loss	AP	Symptom	1	3	Q13
Constipation	CO	Symptom	1	3	Q16
Diarrhea	DI	Symptom	1	3	Q17
Financial difficulties	FI	Symptom	1	3	Q28

The KPS scale refers to the evaluation of a patient’s performance capacity. From another perspective, the KPS scale can assess a patient’s physical condition. The KPS scale is calculated for a total of 100 points, which are partitioned into 10-point increments according to the state of the illness, degree of independent living, and whether or not the patient can perform normal activities. A score of 100 points is consistent with good physical condition without symptoms, 60 points indicates basic self-reliance not requiring assistance, and 0 points is indicative of death [24].

### Safety assessment

The safety assessment is based on version 3.0 of the Common Terminology Criteria for Adverse Events (CTCAE) issued by the National Cancer Institute (NCI) [25]. Liver and kidney function, routine blood, urine, and stool testing, and an electrocardiogram will be inspected to assess drug toxicity and adverse reactions of the two groups.

### Adverse events

According to the CTCAE issued by the NCI, all adverse events (AEs) must be evaluated objectively. If there are patients who enroll in the study and discontinue the study within 30 days due to the emergence of acute and tardive AEs, the details must be recorded and reported. All the AEs after starting the trial should be followed until satisfactory resolution, or the primary researcher should determine if the AEs have stabilized. The relationship between the AE and investigational drugs should be assessed using Table 4 from *Measures for the Reporting and Monitoring of Adverse Drug Reactions*, which is issued by the 2011 Ministry of Health of the People’s Republic of China (Table 4).

**Table 4:** Relevance evaluation standard between AE and investigational drugs

Judgment results	Judgment Index				
	①	②	③	④	⑤
Definite relevance	+	+	--	+	+
Probable relevance	+	+	--	+	?
Difficult to determine	+	+	±	±	?
Probable irrelevance	+	--	±	±	?
Definite irrelevance	--	--	+	--	--

AE Judgment Index: ① Whether or not there is a successive relationship between time of treatment onset and time of AE occurrence. ② Whether or not suspicious AE is in accordance with the known AE of the medicine. ③ Whether or not the suspicious AE can be explained by the effect of combination of medicine, patient’s clinical condition, or other therapies. ④ Whether or not suspicious AE disappears or is relieved after drug withdrawal or dosage reduction. ⑤ Whether or not similar reactions occur after using the suspicious drugs again

+ means affirmative, --means negative, ± means difficult to determine, ? means unclear situation

1- Definite relevance, 2-Probable relevance, 3-Difficult to determine, 4-Probable irrelevance, 5-Definite irrelevance

The incidence of ADR is calculated by using the sum of the cases of 1 + 2 + 3 as numerator, and the number of all cases as denominator

### Serious AEs

If serious AEs occur, including hospitalization, prolonged hospitalization, disability, affect on the ability to work, life endangerment, or death, corresponding countermeasures will be conducted by researchers according to the drug-induced symptoms to ensure patient safety. The results of treatment will be communicated to the project director, the Ethics Committee of Guang’anmen Hospital, and the sponsor of Shanxi Zhendong Pharmaceutical Co., Ltd.

### Data management

According to the subjects’ original observation records, the data will be recorded in a timely and accurate manner into case report forms (CRFs). Supervisors will inspect whether or not the study is carried out as the trial protocol. The CRFs inspected by supervisors will be sent to the data managers after the supervisor’s signature is obtained. Before logging, data managers will check the data again. If problems are found, the supervisor will be informed in a timely manner and the researcher will be asked for the reasons. Data entry clerks will double-enter the data. If any problems exist during the entering procedure, data entry clerks will take the registration and make a report to handle the problem rapidly. Some of the CRFs will be selectively checked after data entry to ascertain the quality of the entry.

The range and logic of data checking will be mapped out according to the range of the index value and the relationship by data managers and primary researchers. The database will be built. The fault data will be controlled before data entry, and the reasons for fault data will be identified and corrected. Fault content and modified results will be recorded and properly preserved.

CRFs will be archived in accordance with the order of the sequence number after data entry and checked. The information will be safely stored to avoid staining, water damage, and crimping. The prescribed period of keeping original files will be in accordance with *Standardizing the quality management of a drug clinical trial*.

### Statistical analysis

SAS statistical software will be used for statistical analysis of the collected data. All of the statistical tests will adopt a two-tailed test, and a significance level of 5 % will be used throughout the analysis.

All of the measurement data for the two groups will undergo descriptive statistics. Both two-sample *t*-tests and Wilcoxon rank sum tests for continuous data and chi-squared tests or Fisher’s exact tests for categorical data will be used to analyze the baseline characteristics. Paired *t*-tests will be used to compare intra-group differences before and after treatment, and variance analysis will be used to compare inter-group data. Kaplan-Meier survival analysis will be used to compare the PFS and OS between the two groups. Log-rank and Cox regression analyses will also be used. For the primary outcome, chi-squared tests will be used to compare the DCRs between the two groups.

### Recruitment

With respect to shedding analyses, chi-squared tests will be used to compare the total shedding rates, including shedding caused by AEs. Regarding efficiency analyses, the primary and secondary indices were analyzed by full analysis set (FAS) and per protocol set (PPS). Because this study is a multicenter clinical trial, the center effect on the indices should be considered. With respect to the safety analysis, chi-squared tests will be used to compare the incidence of AEs, which will be described by tabulation. The laboratory results of changes in testing before and after the trial, and the relationship between the anomaly index and the investigational drugs will be analyzed.

### Ethics

This trial has been reviewed and approved by the Ethics Committee of Guang’anmen Hospital affiliated with the China Academy of Chinese Medical Sciences (approval number: 2014EC086-01). The trial has been registered in the Chinese Clinical Trial Registry (registration number: ChiCTR-IPR-14005484).

## Discussion

CKI, known as the Yanshu injection, is extracted from two Chinese herbs (kushen [*Radix Sophorae flavescentis*] and baituling [*Rhizoma smilacis glabrae*]), consisting of the primary components oxymatrine and matrine [26]. From the perspective of basic experiments, CKI has been shown to be effective in the treatment of cancer with or without combination chemotherapy. Zhang et al. [27] and Xu et al. [28], using cancer stem cells, performed experiments *in vitro* to prove that oxymatrine can directly kill tumor cells and inhibit their proliferation. Li et al. [29], using oxymatrine in combination with chemotherapy, showed that CKI can increase the sensitivity of chemotherapy to kill tumor cells effectively. In addition, Zhao et al. [30] found that CKI blocks TRPV1 signaling and prohibits tumor growth to relieve cancer pain, then improves the patients’ QoL.

Clinically, the positive benefits of CKI in the treatment of lung cancer have also been demonstrated [31]. In the treatment of elderly patients with advanced NSCLC, CKI has been widely used clinically because of its high efficiency and low toxicity. Lu et al. [32] performed a systematic review on CKI adjuvant chemotherapy in the treatment of lung cancer. The review involved 23 RCTs (1,750 cases). The results showed that the tumor objective response rate (OR = 1.68, 95 % CI = 1.32–2.04, *P* < 0.0001) and QoL (OR = 2.69, 95 % CI = 1.97–3.68, *P* < 0.00001) in patients who received CKI adjuvant chemotherapy and chemotherapy alone favored the former. CKI has been shown to be a safe drug. Additionally, Qin [33] enrolled 86 elderly patients who were randomly divided into an experimental group (*n* = 43), who received CKI in combination with chemotherapy (docetaxel with carboplatin), and a control group (*n* = 43), who received the docetaxel with carboplatin chemotherapy alone. Though no statistically significant difference existed between the two groups in efficacy (55.81 % versus 53.49 %, *P* > 0.05), the incidence of adverse reactions, including nausea and vomiting, fatigue, constipation, and arrest of bone marrow, was lower in the experimental group than in the control group (*P* < 0.05). The above-mentioned studies could provide evidence for the feasibility of this trial.

The ages of the targets in this trial will be > 65 years, in accordance with the definition of the elderly issued by WHO and the Chinese constitution. Patients > 75 years of age may not tolerate platinum-based doublet chemotherapy and may violate medical ethics. Therefore, the ages of the targets will be < 75 years.

There are still some limitations and shortcomings in the trial design. First, the chemotherapy regimens are different. Although many studies have demonstrated that these chemotherapy regimens have similar efficacy in patients with NSCLC [17, 19], the ADRs of these regimens are different. Thus, when performing the statistics for the ADRs at the end of the trial, a hierarchical analysis will be performed according to the different chemotherapy regimens. In the future, we will redesign a single-regimen study based on the results of this trial. Second, classification of NSCLC pathologic type is not further analyzed. Third, the requirement on the ages means this trial cannot cover elderly patients > 75 years of age. Consequently, in the future we need to conduct the study further based on what we obtain from this designed trial to clarify the range of this method and to redesign a new trial for use in the elderly > 75 years of age.

This paper summarized the methodology for a multicenter, randomized, controlled, open-label study of CKI in combination with single-agent chemotherapy in elderly patients with advanced NSCLC that refers to the CONSORT statement [34]. The results of this study will support and provide parameters for a novel therapy (CKI in combination with single-agent chemotherapy) used in the treatment of elderly patients with advanced NSCLC.

## Trial status

The trial is currently open to recruitment.

## Abbreviations

ADR: adverse drug reaction
AE: adverse event
CKI: compound kushen injection
CR: complete response
CRF: case report form
CRO: contract research organization
KPS: Karnofsky Performance Status
NCI: National Cancer Institute
NSCLC: non-small cell lung cancer
OS: overall survival
PD: progressive disease
PFS: progression-free survival
PR: partial response
QoL: quality of life
RECIST: Response Evaluation Criteria in Solid Tumors
SD: stable disease
TCM: traditional Chinese medicine
